# Effectiveness of Audio-Tactile Performance Versus Other Oral Health Education Methods in Improving Oral Health in Visually Impaired Children and Adolescents: A Systematic Review

**DOI:** 10.7759/cureus.66708

**Published:** 2024-08-12

**Authors:** Mahalakshmi Kumaraguru, Srisakthi D, Monal Yuwanati, Meignana Arumugham I

**Affiliations:** 1 Public Health Dentistry, Saveetha Dental College and Hospitals, Saveetha Institute of Medical and Technical Sciences, Saveetha University, Chennai, IND; 2 Oral Pathology and Microbiology, Saveetha Dental College and Hospitals, Saveetha Institute of Medical and Technical Sciences, Saveetha University, Chennai, IND

**Keywords:** education, oral health, children, braille, performance, tactile, audio, visually impaired, blind

## Abstract

The aim of this study was to compare the effectiveness of audio-tactile performance (ATP) versus other oral health education methods in improving the oral health status of visually challenged children and adolescents. The review was carried out based on the Preferred Reporting Items for Systematic Reviews and Meta-Analyses (PRISMA) guidelines. A systematic search was performed on the effectiveness of ATP in improving the oral health of visually impaired children. The search was conducted in Scopus, Google Scholar, PubMed, Embase, Lilacs, Web of Science, and Cochrane until December 2022. The risk-of-bias tool for randomized trials (RoB-2) was used to analyze the risk of bias. Meta-analysis was carried out for included studies that made similar comparisons and reported the same outcome measures. The initial search retrieved 368 records, of which nine studies were included for qualitative synthesis. Out of the nine included studies, five studies were included for quantitative synthesis. Two studies evaluating plaque index at 180 days (MD = -0.10; p = 0.0009; CI = -0.17 to -0.02) and five studies evaluating gingival index at 90 days (MD = -0.15; p < 0.00001; CI= -0.21 to -0.09) exhibited a significant mean difference favoring ATP. Three studies that evaluated gingival index at 30 days and 180 days showed significant mean differences (MD = -0.27; p < 0.000; CI = -0.40 to -0.15 and MD = -0.09; p = 0.01; CI= -0.15 to -0.02) favoring ATP. The ATP technique produced significant improvements in oral health when compared with other conventional techniques. However, the studies had high heterogeneity, and hence, the result must be inferred with caution.

## Introduction and background

At least 210,000 children in India have severe visual impairment (SVI) or are blind, with the prevalence of childhood blindness reported to be 0.5/1,000. Almost half of the causes of the estimated 15,000 students in schools for the blind are preventable [1].

Previous research has proven that poor oral hygiene, dental caries [2], different stages of periodontal diseases [3,4], trauma to the anterior teeth [5], and hypoplastic teeth are highly prevalent in visually challenged children. This, in turn, leads to a poor quality of life characterized by high rates of absenteeism from school and difficulties in eating, sleeping, and leading a normal social life. To combat the abovementioned problems, it is necessary to improve the oral health of visually impaired children [6].

Visually impaired children are unable to visualize plaque and have poor hand-eye coordination, so their manual dexterity and plaque removal efficiency are limited. These are the primary causes of their poor oral health [2]. Other reasons include sparse dental visits, barriers to utilizing oral health care, and disregard from parents and dental professionals. At the same time, the prime goal is directed toward handling their existing disability [1].

According to research conducted by Khurana et al., a considerable proportion of the children (35.75%) who were visually impaired did not know how to take care of their teeth, but after participating in a dental health education program, 77.57% of the study population reported the frequency of tooth brushing to be two times in a day [7]. In research carried out by Nandini et al. [8], only a mere 10.67% of visually challenged children brushed their teeth twice a day without receiving any dental health education.

Effectively transmitted oral health education (OHE) contributes significantly to the improvement of various oral health problems and nurtures the relationship between blind individuals and dentists. However, the visually impaired cannot learn brushing techniques by visual imitation. They navigate the external world by means of their other senses such as touch and hearing. Multiple and varied tailor-made methods that garner the benefits of these other senses for imparting satisfactory OHE to the visually impaired, like audio aids, OHE instructions in the form of Braille booklets, use of large-size tooth models, and audio-tactile performance (ATP) exist [9].

Among these, Braille is the most commonly used method in routine health education. Braille script consists of raised dots arranged in a particular manner to depict various alphabets, which the reader feels using their fingers. However, this method can be time-consuming, monotonous, and cumbersome for many, especially younger children. Audio aids, on the other hand, can be time-saving, cost-effective, and can be played repeatedly in the form of instructions or music [10].

ATP is a multisensory method of health education that uses audio aids (audio component) and tactile aids (tactile component) to educate children, followed by the child's active participation (performance component) in demonstrating the technique learned [9]. These multiple components have a synergistic effect on each other, thus enhancing the efficiency of learning. Moreover, the use of multiple components will also hold the attention of the child, as children's minds are inclined toward variety, creativity, and novelty [10].

The untapped potential of innovative and novel OHE in molding a child’s oral health practices and knowledge is a topic subject to much dispute as there is a dearth of information from research on this topic, especially in children with special needs such as those with visual impairment [11]. Though a recent systematic review [9] did assess the effectiveness of ATP, a quantitative analysis was not done, thus limiting the quality of evidence. The aim of this systematic review was to compare the effectiveness of ATP versus other OHE methods in improving the oral health of visually impaired children and adolescents. The objective of this systematic review was to conduct a subgroup analysis to compare the effectiveness of ATP and other OHE methods at different follow-up time periods.

## Review

Protocol

This systematic review and meta-analysis followed the Preferred Reporting Items for Systematic Reviews and Meta-Analyses (PRISMA) 2020 guidelines [12].

Review question

Is ATP more effective than Braille, audio, tactile, and other conventional methods of OHE in inculcating oral health behavior among visually impaired children?

Studies selection criteria

Type of Studies

All prospective human trials, including randomized controlled trials, clinical trials, and non-randomized trials, from January 2013 to June 2023, were included in this systematic review and meta-analysis.

Type of Participants

Visually impaired individuals of age ranging from six to 17 years in any setting requiring OHE to maintain their oral health were considered.

Type of Interventions

OHE by ATP method compared with other methods such as Braille, audio, tactile, and conventional methods.

Types of Outcome Measures

Primary outcome: The outcome of the present systematic review and meta-analysis is the reduction in the oral hygiene index simplified (OHI-S), plaque index (PI), and gingival index (GI) scores.

Secondary outcomes: The secondary outcome included is the percentage of improvement in oral health knowledge among the participants.

Source and search strategy

Electronic Searches

The following are the databases considered to identify the studies to be included in this review: PubMed, Cochrane Library, Scopus, Web of Science, Google Scholar, EMBASE, and trial registries. The search terms and MeSH terms used for search following the PICO principle are presented in Table 1.

**Table 1:** Search strategy SCI-EXPANDED: Science Citation Index - Expanded; CPCI-S: Conference Proceedings Citation Index - Science; ESCIS: Emerging Sources Citation Index

Search engine	Search terms
PubMed (96 results)	("visually impaired children" (All Fields) OR "visually impaired adolescents" (All Fields) OR "visually challenged individuals" (All Fields) OR "visually challenged people" (All Fields) OR "visually challenged subjects" (All Fields) OR "visually impaired persons" (MeSH Terms)) AND ("audio tactile performance" (All Fields) OR "audio tactile practice" (All Fields) OR "audio tactile performance technique" (All Fields)) AND ("audio education" (All Fields) OR "tactile education" (All Fields) OR "braille education" (All Fields) OR "conventional health education" (All Fields) OR "conventional health education methods" (All Fields) OR "oral health/education" (MeSH Terms)) AND ("oral health knowledge" (All Fields) OR "oral health knowledge and attitudes" (All Fields) OR "oral health knowledge and behavior" (All Fields) OR "oral health knowledge and oral health attitudes" (All Fields) OR "oral health knowledge scores" (All Fields) OR "oral health knowledge rate" (All Fields) OR "oral health knowledge test" (All Fields) OR "oral hygiene index" (All Fields) OR "oral hygiene index simplified" (All Fields) OR "Silness plaque index" (All Fields) OR "gingival index" (All Fields) OR "Silness gingival indices" (All Fields) OR "plaque indices" (All Fields))
Cochrane Library (73 results)	#1 ("visually impaired children") OR ("visually impaired adolescents") OR ("visually challenged individuals") OR ("visually challenged people") OR ("visually challenged subjects") (Word variations have been searched)
	#2 MeSH descriptor: (visually impaired persons) explode all trees
	#3 #1 OR #2
	#4 ("audio tactile performance") OR ("audio tactile practice") OR ("audio tactile performance technique")
	#5 ("audio education") OR ("tactile education") OR ("braille education") OR ("conventional health education") OR ("conventional health education methods")
	#6 MeSH descriptor: (oral health education) explode all trees
	#7 #5 OR #6
	#8 ("oral health knowledge") OR ("oral health knowledge and attitudes") OR ("oral health knowledge and behavior") OR ("oral health knowledge and oral health attitudes") OR ("oral health knowledge scores") OR ("oral health knowledge rate") OR ("oral health knowledge test") OR ("oral hygiene index") OR ("oral hygiene index simplified") OR ("Loe plaque index") OR ("Silness plaque index") OR ("gingival index") OR ("Silness gingival index") OR ("Loe gingival index")
	#9 #3 AND #4 AND #7 AND #8
Scopus (56 results)	(TITLE-ABS-KEY (“visually impaired children” OR “visually impaired adolescents” OR “visually challenged individuals” OR “visually challenged people” OR “visually challenged subjects”)) AND (TITLE-ABS-KEY (“audio tactile performance” OR “audio tactile practice” OR “audio tactile performance technique”)) AND (TITLE-ABS-KEY (“audio education” OR “tactile education” OR “braille education” OR “conventional health education” OR “conventional health education methods)) AND (TITLE-ABS-KEY (“Oral health knowledge” OR “oral health knowledge and attitudes” OR “oral health knowledge and behavior” OR “oral health knowledge and oral health attitudes” OR “oral health knowledge scores” OR “oral health knowledge rate” OR “oral health knowledge test” OR “oral hygiene index” OR “oral hygiene index” OR “oral hygiene index simplified” OR “Loe plaque index” OR “Silness plaque index” OR “gingival index” OR “Silness gingival index” OR “Loe gingival index”))
Web of Science (45 results)	# 5
	#4 AND #3 AND #2 AND #1
	Indexes = SCI-EXPANDED, CPCI-S, ESCI; timespan = 10 years (2013-2023)
	# 4
	(ALL = (Oral health knowledge OR oral health knowledge and attitudes OR oral health knowledge and behavior OR oral health knowledge and oral health attitudes OR oral health knowledge scores OR oral health knowledge rate OR oral health knowledge test OR oral hygiene index OR oral hygiene index OR oral hygiene index simplified OR Loe plaque index OR Silness plaque index OR gingival index OR Silness gingival index OR Loe gingival index)) AND language: (All) AND document types: (Article)
	Indexes = SCI-EXPANDED, CPCI-S, ESCI; timespan = 10 years (2013-2023)
	# 3
	(ALL = (audio education OR tactile education OR braille education OR conventional health education OR conventional health education methods)) AND language: (All) AND document types: (Article)
	Indexes = SCI-EXPANDED, CPCI-S, ESCI; timespan = 10 years (2013-2023)
	# 2
	(ALL = (audio tactile performance OR audio tactile practice OR audio tactile performance technique)) AND language: (All) AND document types: (Article)
	Indexes = SCI-EXPANDED, CPCI-S, ESCI; timespan = 10 years (2013-2023)
	# 1
	(ALL = (visually impaired children OR visually impaired adolescents OR visually challenged individuals OR visually challenged people OR visually challenged subjects)) AND language: (All) AND document types: (Article)

Search for Other Resources

A hand search, with the help of a librarian, was carried out in community dentistry journals, public health journals, oral epidemiology journals, dental health journals, preventive care journals, relevant conference proceedings, and trial registries for any ongoing trials. No language restrictions were placed. Trials carried out in the past 10 years (2013-2023) were considered.

Data collection and analysis

Selection of Studies

Rayyan (Qatar Computing Research Institute, Qatar) software, an artificial intelligence tool for systematic review, was used to remove the duplicate studies obtained from different databases. Screening for title and abstract was also carried out in Rayyan software by two reviewers individually for inclusion criteria, explaining the reason for exclusion. Any consensus on screening for the title and abstract has been resolved by the third reviewer. Following this, the full text of the remaining studies was independently evaluated for eligibility criteria by the two reviewers. Again, any lack of consensus was brought to the notice of the third reviewer and resolved.

Data Extraction and Management

Data extraction was carried out by two reviewers individually using customized data extraction forms. These data extraction forms were pilot-tested with a few papers, and required modifications were done before use. Any disagreement in data extraction forms was discussed and resolved by the third reviewer. Respective study authors were contacted for any missing information. Data were not included if further information was not obtained. The data recorded for each included trial were author, year of publication, country where the trial was carried out, type of study (randomized or non-randomized), participants' demographic details and criteria for inclusion, intervention (ATP and Braille, audio, tactile and conventional methods) type and relevant details, outcome details including method of assessment, and follow-up details.

Assessment of risk of bias

The assessment of the quality of the included studies was undertaken by two reviewers independently as a part of the data extraction process. The two reviewers who assessed for the risk of bias were not blinded to the authors of the included studies. The revised Cochrane risk-of-bias tool for randomized trials (RoB-2) [11] was used to analyze the randomized studies, and the Risk of Bias in Non-randomized Studies - of Interventions (ROBINS-I) tool was used to analyze non-randomized studies [12]. RoB-2 was used to assess the included studies by evaluating five domains.

Data synthesis

The continuous outcome measures from each included study were pooled and summarized as mean differences and standard deviations to represent the effect of an intervention. Included studies with similar comparisons reporting the same outcome measures were carried onto meta-analysis. The weighted mean difference for continuous data using inverse variance and fixed effects model (mentioned as FE in the results section) were obtained to result in conservative estimates of the confidence interval. Any significant discrepancies in the estimates of treatment effects from different studies were assessed with the I^2^ test for heterogeneity. I^2^ > 40% was considered as high heterogeneity. A funnel plot was used to assess publication bias across the included studies.

Subgroups and sensitivity analysis

We the reviewers intended to carry out a subgroup analysis with plaque and GI for the period of follow-up. However, only few studies rendered themselves suitable to carry out sub group analysis. Also, a sensitivity analysis was carried out to examine the effect of randomization, allocation concealment, and blinding for outcome assessment on overall effect estimation.

Quality of evidence assessment

The Grading of Recommendations Assessment, Development, and Evaluation (GRADE) approach was used to assess the quality of evidence from the meta-analysis. GRADEpro GDT software (Evidence Prime, Hamilton, Ontario, CA) was used with results that indicated very low, low, moderate, or high quality of evidence.

Results

Study Search and Selection

**Figure 1:**
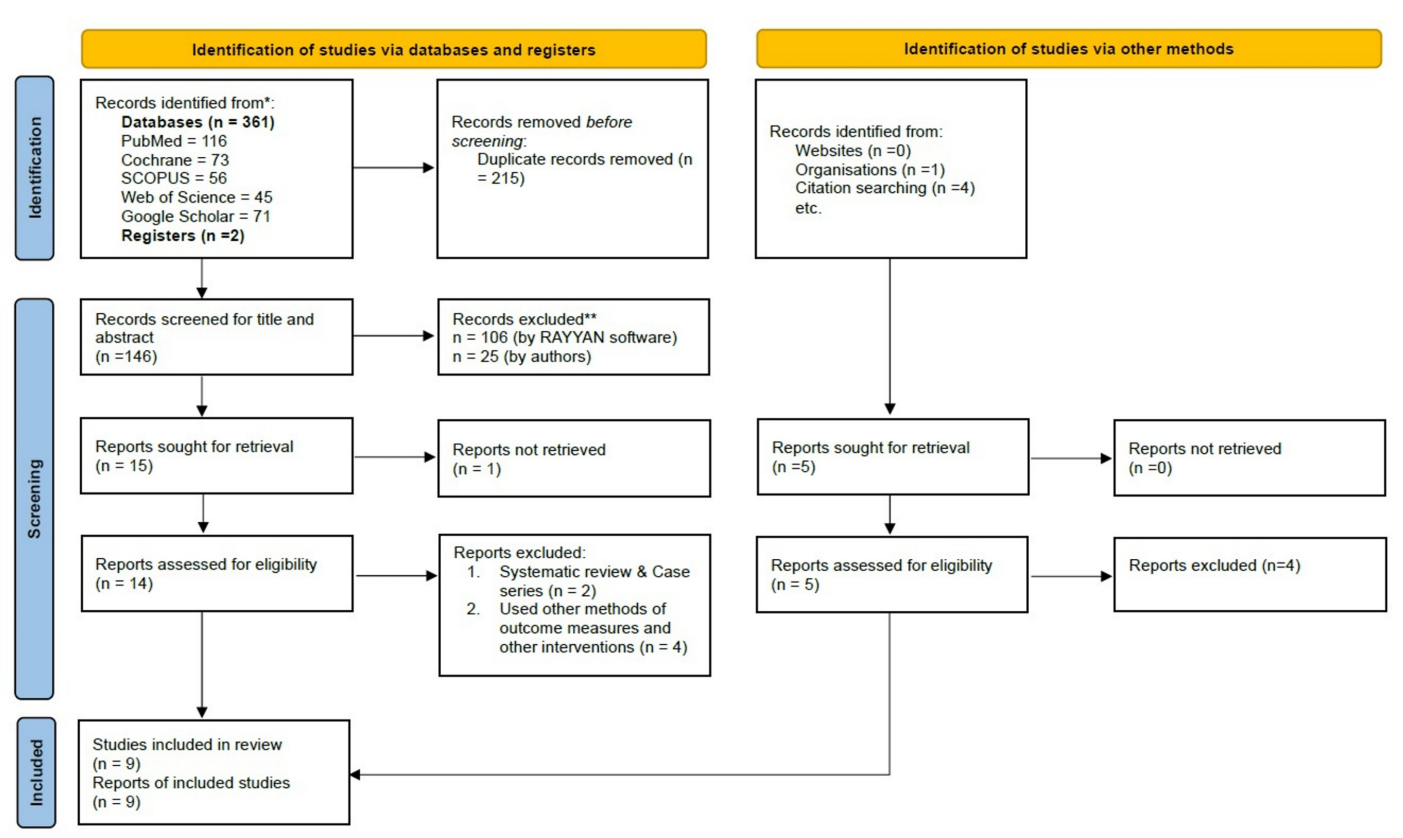
Preferred Reporting Items for Systematic Reviews and Meta-Analyses (PRISMA) 2020 flow diagram showing the screening and selection process for identifying the relevant studies for the present systematic review

A total of 368 studies were obtained on an initial search through PubMed, Cochrane Library, Scopus, Web of Science, Google Scholar, EMBASE, hand search, trial registries, and other methods such as organizations and citing searching. After removing 215 duplicate studies, 148 studies were screened for title and abstract using Rayyan software. About 137 studies not suitable for this systematic review were excluded. Of the remaining 15 studies, full reports of one study were not retrieved after trying to contact the author. Thus, full reports of 14 studies from database search and five reports from other method search were screened for eligibility (Figure 1). Two systematic reviews, one umbrella review, one pre- and post-intervention with no comparison, five studies compared other methods with no ATP, and one study used patient performance index as an outcome measure were excluded, given in Table 2 with reasons. Finally, nine studies were included for qualitative synthesis [13-21], out of which five studies were included for quantitative synthesis.

**Table 2:** List of excluded studies with reason

Author/year	Reason for exclusion
Ganapathi et al., 2015	Compared various sensory methods but not ATP.
Qureshi et al., 2017	Compared tactile with verbal, oral health message.
Sardana et al., 2019	Compared tactile and audio methods separately.
Sharfifard et al., 2020	ATP was in the control group, test group had ATP, game combinations.
Masoumi et al., 2021	Compared verbal tactile with verbal braille but not audio tactile.
Deshpande et al, 2023	Systematic review comparing all methods of oral health education.
Bhor et al., 2020	Systematic review comparing all methods of oral health education.
Deolia et al., 2019	Comparison of pre and post-ATP with no comparison group.
Sardana et al., 2023	Oral health education for visual impairment - umbrella review.
Widodo et al., 2023	Patient performance index is the outcome measure.

Characteristics of the Included Studies

The characteristics of the nine included studies are presented in Table 3 and Table 4. A total of 959 children, ages ranging from six to 18 years, were evaluated for plaque, gingival, oral hygiene simplified index, oral health knowledge with ATP, and other methods of OHE. Braille method of OHE was the commonly used method for comparison with ATP. About six comparison studies evaluated the effectiveness of OHE using Silness and Loe PI [13,15-19], and six comparison studies used Loe and Silness GI [14-18,20] for evaluation. Six studies had combinations of ATP as one group in their comparison [13,16-20]. Among nine studies, randomized study design was used by eight studies [13-15,17-21], whereas one study conducted a non-randomized trial [16].

**Table 3:** Characteristics of included studies - author, study design, age of participants, sample size, and groups

Author/year	Study design	Sample size	Age range	Oral health education groups (n)
Deshpande et al., 2017 [4]	Randomized trial	60 children	12-16 years	Group 1 (20): received Braille; group 2 (20): received audio tactile performance (ATP); group 3 (20): received combination of Braille and ATP
Das et al., 2019 [5]	Randomized controlled trial	60 children	10-15 years	Test group (30): received ATP; control group (30): received Braille and audio
Sriram et al., 2019 [6]	Randomized clinical trial	112 children	10-17 years	Braille group (56); ATP group (56)
Tiwari et al., 2019 [7]	Non-randomized interventional study	90 children	12-15 years	Group 1 (30): ATP; group 2 (30): Braille; group 3 (30): ATP + Braille
Gautam et al., 2020 [8]	Randomized controlled trial	180 children	9-17 years	Group 1 (60): Braille; group 2 (60): ATP; group 3 (60): ATP + Braille (BATP)
Indurkar et al., 2021 [9]	Randomized interventional study	51 children	9-15 years	Group 1 (17): Braille; group 2 (17): ATP; group 3 (17): ATP + Braille (BATP)
Nair et al., 2021 [10]	Randomized interventional study	90 children	6-15 years	Group 1 (30): audio method (AM); group 2 (30): ATP; group 3 (30): ATP + Braille
Shrivatsava et al., 2022 [11]	Randomized clinical trial	96 children	6-16 years	Group 1 (32): verbal; group 2 (32): Braille; group 3 (32): AATP
Sowmya et al., 2022 [12]	Randomized interventional study	220 children	8-18 years	Group 1 (110): Braille; group 2 (110): ATP

**Table 4:** Characteristics of included studies - outcome, results, and inferences

Author/year	Outcomes measured	Results			Inference
		Follow-up	Group	Mean ± SD/mean difference ± SD	
Deshpande et al., 2017 [4]	Loe plaque index (1967)	Not mentioned			Combination of Braille and ATP was most effective
Das et al., 2019 [5]	Oral health knowledge; Loe and Silness gingival index (1963) - Mean ± SD	Baseline	Test	4.58 ± 1.63	ATP was better than the combination of braille and audio
			Control	4.12 ± 1.66	
		30 days	Test	1.23 ± 2.43	
			Control	0.92 ± 2.52	
		90 days	Test	2.65 ± 1.64	
			Control	1.97 ± 1.48	
Sriram et al., 2019 [6]	Silness and Loe plaque index (1964) - Mean ± SD	Baseline	Braille	1.72 ± 0.15	Braille and ATP improved oral health of visually impaired children
			ATP	1.71 ± 0.15	
		90 days	Braille	1.11 ± 0.30	
			ATP	1.12 ± 0.26	
	Loe and Silness gingival index (1963) - Mean ± SD	Baseline	Braille	0.85 ± 0.45	
			ATP	0.85 ± 0.31	
		90 days	Braille	0.65 ± 0.31	
			ATP	0.57 ± 0.15	
Tiwari et al., 2019 [7]	Silness and Loe plaque index (1964) - Mean ± SD	Baseline	Group 1	1.68 ± 0.26	Combination of ATP and Braille is an effective way to improve oral hygiene status
			Group 2	1.70 ± 0.29	
			Group 3	1.74 ± 0.29	
		90 days	Group 1	1.15 ± 0.16	
			Group 2	1.40 ± 0.24	
			Group 3	1.01 ± 0.20	
	Loe and Silness gingival index (1963) - Mean ± SD	Baseline	Group 1	1.78 ± 0.25	
			Group 2	1.81 ± 0.29	
			Group 3	1.84 ± 0.29	
		90 days	Group 1	1.25 ± 0.15	
			Group 2	1.50 ± 0.24	
			Group 3	1.11 ± 0.19	
Gautam et al., 2020 [8]	Silness and Loe plaque index (1964) - Mean ± SD	Baseline	Group 1	1.67 ± 0.51	Acceptable level of oral health can be maintained using a combination of Braille and ATP
			Group 2	1.84 ± 0.43	
			Group 3	1.84 ± 0.38	
		90 days	Group 1	1.16 ± 0.43	
			Group 2	0.95 ± 0.32	
			Group 3	0.80 ± 0.27	
	Loe and Silness gingival index (1963) - Mean ± SD	Baseline	Group 1	1.69 ± 0.48	
			Group 2	1.87 ± 0.38	
			Group 3	1.85 ± 0.33	
		90 days	Group 1	1.21 ± 0.45	
			Group 2	1.00 ± 0.32	
			Group 3	0.79 ± 0.18	
Indurkar et al., 2021 [9]	Silness and Loe plaque index (1964) - Mean ± SD	Baseline	Group 1	2.17 ± 0.81	Combination of Braille and ATP are more effective
			Group 2	1.88 ± 0.78	
			Group 3	2.10 ± 0.75	
		90 days	Group 1	1.81 ± 1.13	
			Group 2	1.57 ± 0.81	
			Group 3	1.47 ± 0.72	
	Loe and Silness gingival index (1963) - Mean ± SD	Baseline	Group 1	1.24 ± 1.09	
			Group 2	0.94 ± 0.97	
			Group 3	1.57 ± 1.18	
		90 days	Group 1	1.00 ± 0.94	
			Group 2	0.71 ± 0.84	
			Group 3	0.36 ± 0.44	
Nair et al., 2021 [10]	Silness and Loe plaque index (1964) - Mean difference ± SD	90 days	Group 1	0.24 ± 0.26	Combination of Braille with ATP was most effective
			Group 2	0.65 ± 0.24	
			Group 3	1.12 ± 0.26	
		180 days	Group 1	0.03 ± 0.11	
			Group 2	0.74 ± 0.32	
			Group 3	1.23 ± 0.26	
Shrivatsava et al., 2022 [11]	Loe and Silness gingival index (1963) - Mean ± SD	Baseline	Group 1	1.48 ± 0.62	Combination of verbal, Braille, and ATP methods is recommended
			Group 2	1.56 ± 0.56	
			Group 3	1.51 ± 0.50	
		90 days	Group 1	1.32 ± 0.44	
			Group 2	1.28 ± 0.35	
			Group 3	1.29 ± 0.41	
		180 days	Group 1	0.67 ± 0.22	
			Group 2	0.44 ± 0.15	
			Group 3	0.65 ± 0.31	
Sowmya et al., 2022 [12]	Oral hygiene index - simplified (OHI-S) (1964) - Mean ± SD	Baseline	Group 1	1.83 ± 0.68	ATP is better than Braille
			Group 2	1.79 ± 0.67	
		270 days	Group 1	1.80 ± 0.66	
			Group 2	1.75 ± 0.65	

Methodological Quality Assessment of Included Studies

**Figure 2:**
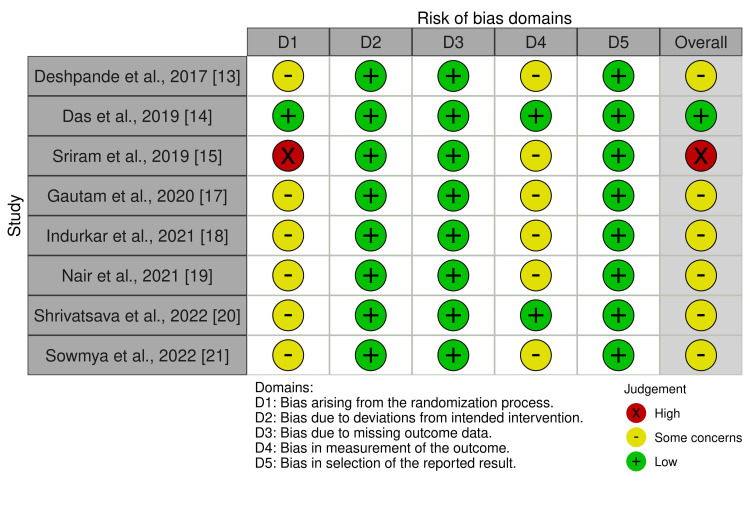
Risk of bias graph (risk-of-bias tool for randomized trials (RoB-2))

**Figure 3:**
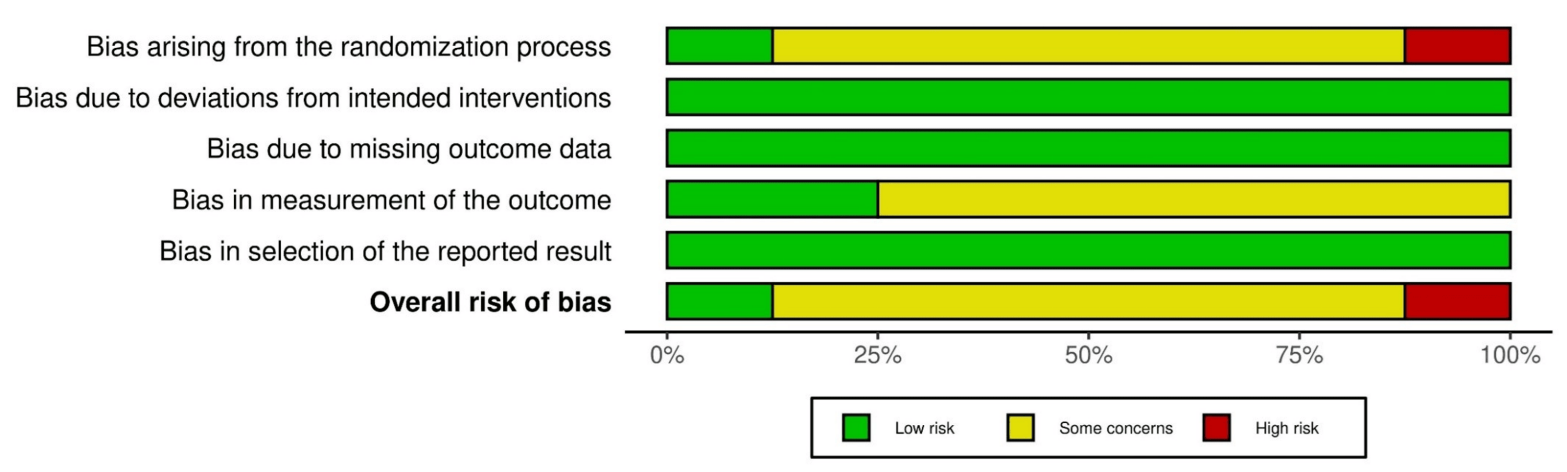
Risk of bias summary graph (risk-of-bias tool for randomized trials (RoB-2))

**Figure 4:**
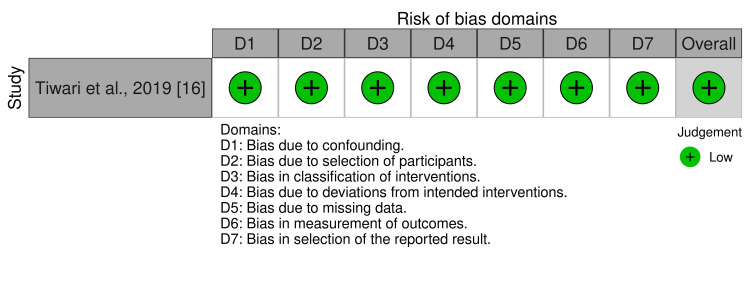
Risk of bias graph (Risk of Bias in Non-randomized Studies - of Interventions (ROBINS-I))

The quality assessment for randomized controlled trials assessed using RoB-2 is presented as risk of bias and risk of bias summary graph in Figure 2 and Figure 3. Similarly, the quality assessment for non-randomized trials assessed using the ROBINS-I tool is presented as a risk of bias in Figure 4. The results of the evaluation of eight randomized trials by the RoB-2 tool showed that one study had a high risk of bias [15], six studies had some concerns [13,16-21], and one study had a low risk of bias [14]. A high risk of bias was found in the bias arising from the randomization process domain [15]; six studies had some concerns about bias arising from the randomization domain and bias in the measurement of the outcome [13,17-21]. Also, the result of one non-randomized trial evaluated by the ROBINS-I tool showed a low risk of bias in all domains [15].

Meta-Analyses

**Figure 5:**
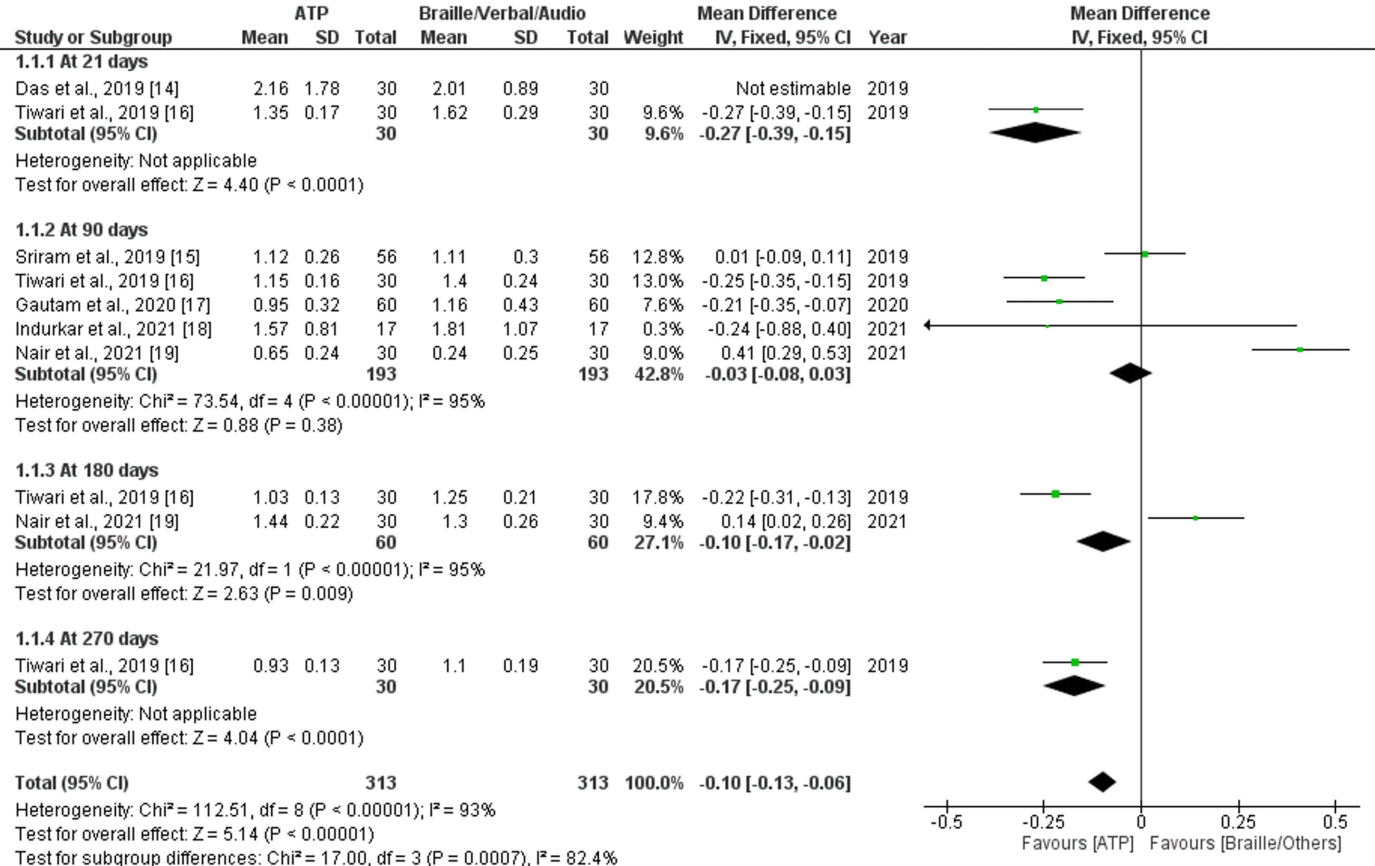
Forest plot showing pooled mean and SD of plaque index at different follow-up periods (significant mean difference observed)

**Figure 6:**
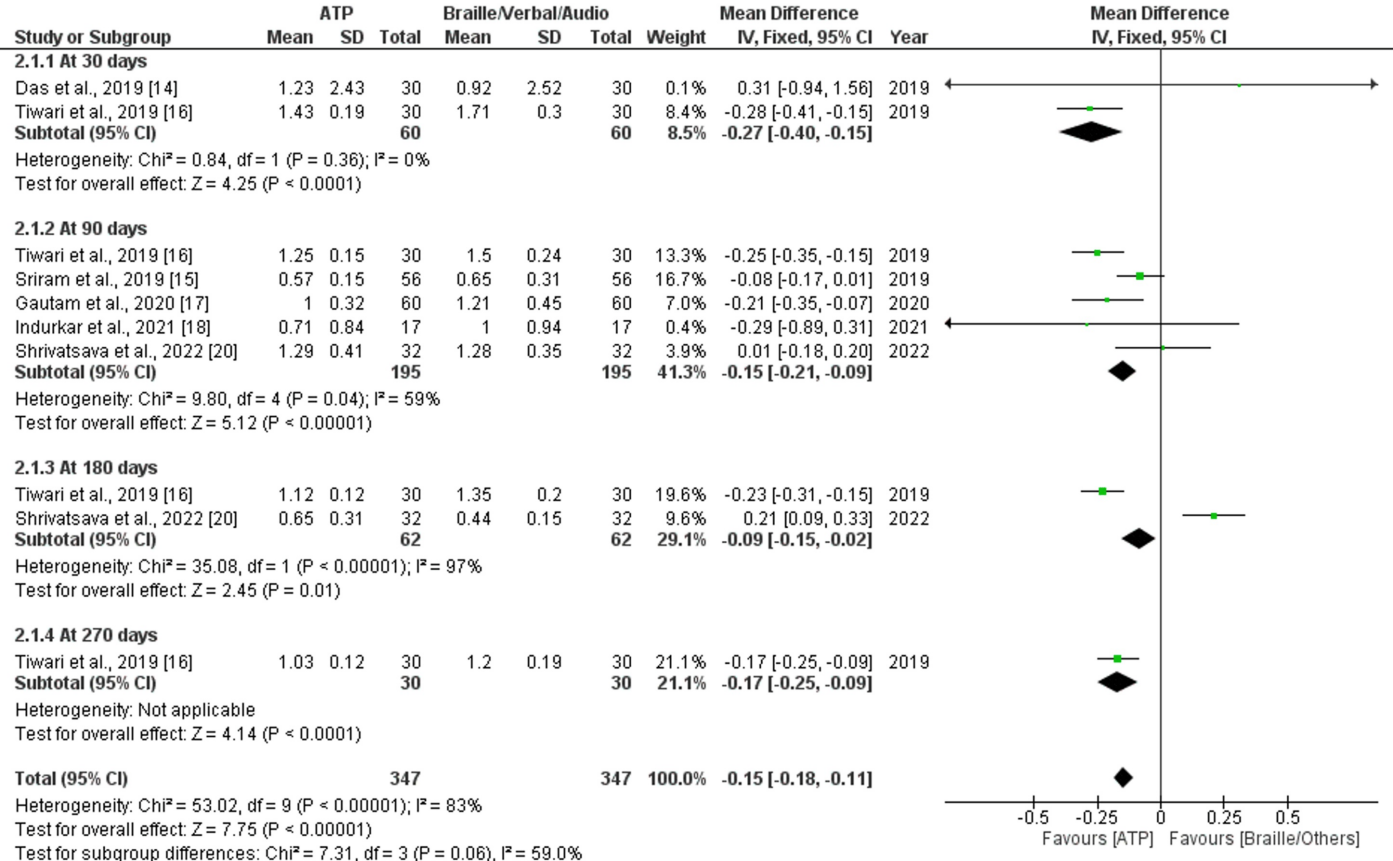
Forest plot showing pooled mean and SD of gingival index at different follow-up periods (significant mean difference observed)

A total of five studies that assessed OHE by PI and gingival index to compare the effect of ATP and other methods (Braille, audio, tactile) were included for meta-analysis. Comparison of ATP combinations with other methods (Braille, audio, tactile) was not included in the meta-analysis. Data were pooled for plaque and GI with similar follow-up periods and a similar outcome presentation method (mean and SD), and forest plots were produced accordingly. Forest plots of five studies, which assessed PI at 90 days, showed no significant mean difference between ATP and other methods with a high amount of heterogeneity (p = 0.38; I^2^ = 95%) (Figure 5). Two studies that assessed PI at 180 days showed a significant mean difference (MD = -0.10; p = 0.0009; CI = -0.17 to -0.02) favoring the ATP method. Forest plots of five studies, which assessed GI at 90 days, showed a significant mean difference (MD = -0.15; p < 0.00001; CI = -0.21 to - 0.09), favoring the ATP method with a moderate amount of heterogeneity (I^2^ = 59%). Three studies at 30 days and 180 days, which assessed GI, showed significant mean difference (MD = -0.27; p < 0.0001; CI = -0.40 to - 015 and MD = -0.09; p = 0.01; CI = -0.15 to -0.02) favoring ATP with low (I^2^ = 0%) and high (I^2^ = 97%) amount of heterogeneity, respectively. The risk in both interventions had a moderate amount of heterogeneity (I^2^ = 22%) (Figure 6).

Sub-Group Analysis

A sub-group analysis of PI at 21 days, 90 days, 180 days, and 270 days reported an overall effect of significant mean difference (MD = -0.10; p < 0.00001; CI = -0.13 to -0.06) with a high amount of heterogeneity (I^2^ = 93%) favoring ATP method (Figure 5). Similarly, sub-group analysis of GI at 30 days, 90 days, 180 days, and 270 days reported an overall effect of significant mean difference (MD = -0.15; p < 0.00001; CI = -0.18 to -0.11 with high amount heterogeneity (I^2^= 83%), favoring ATP method (Figure 6).

Sensitivity Analysis

The elimination of studies with a high methodological risk of bias showed no significant difference in forest plots in the outcomes assessed for sensitivity analysis.

Publication Bias

**Figure 7:**
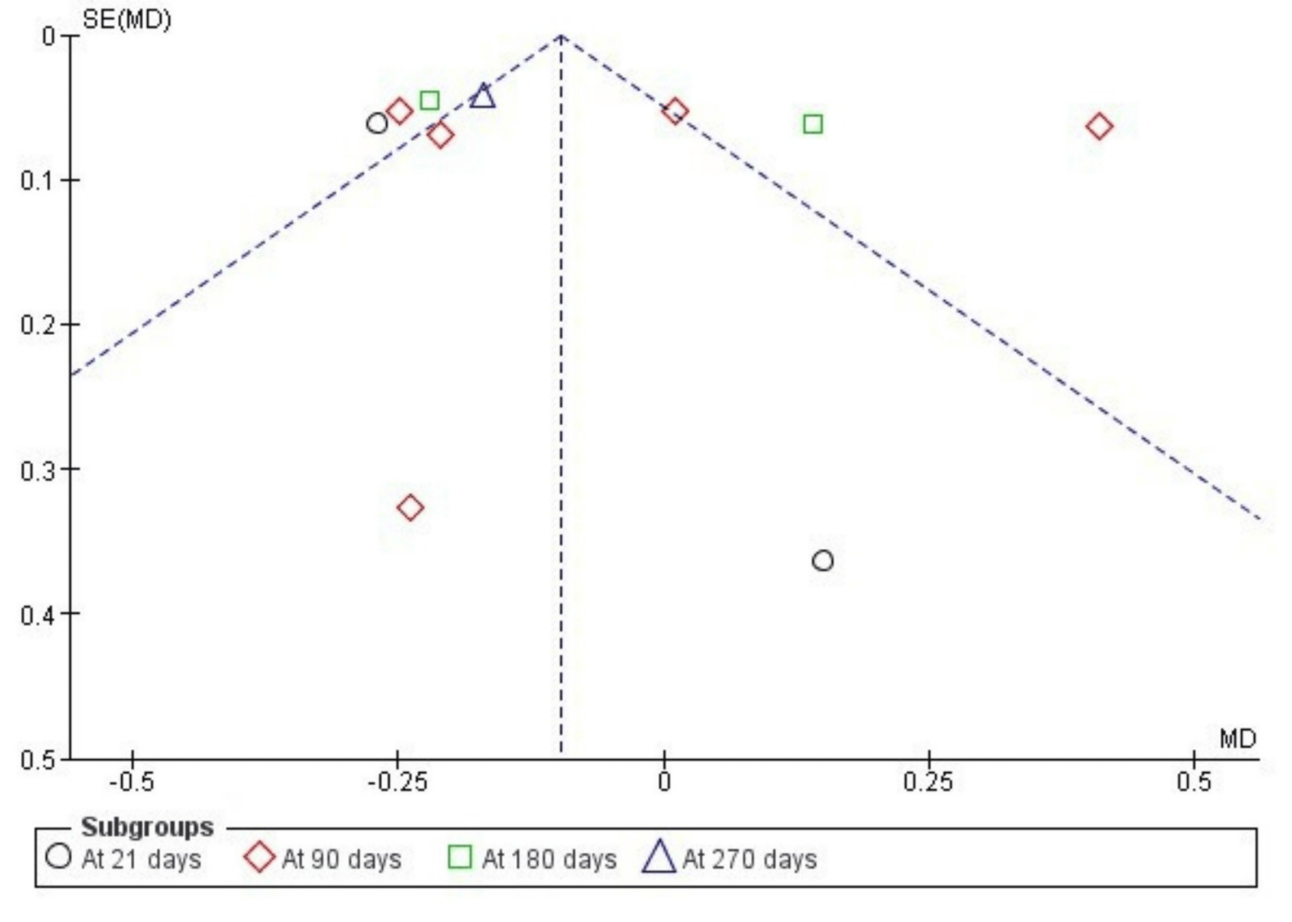
Funnel plot showing publication bias in the assessment of plaque index (indicating studies with high standard error and wide confidence interval)

**Figure 8:**
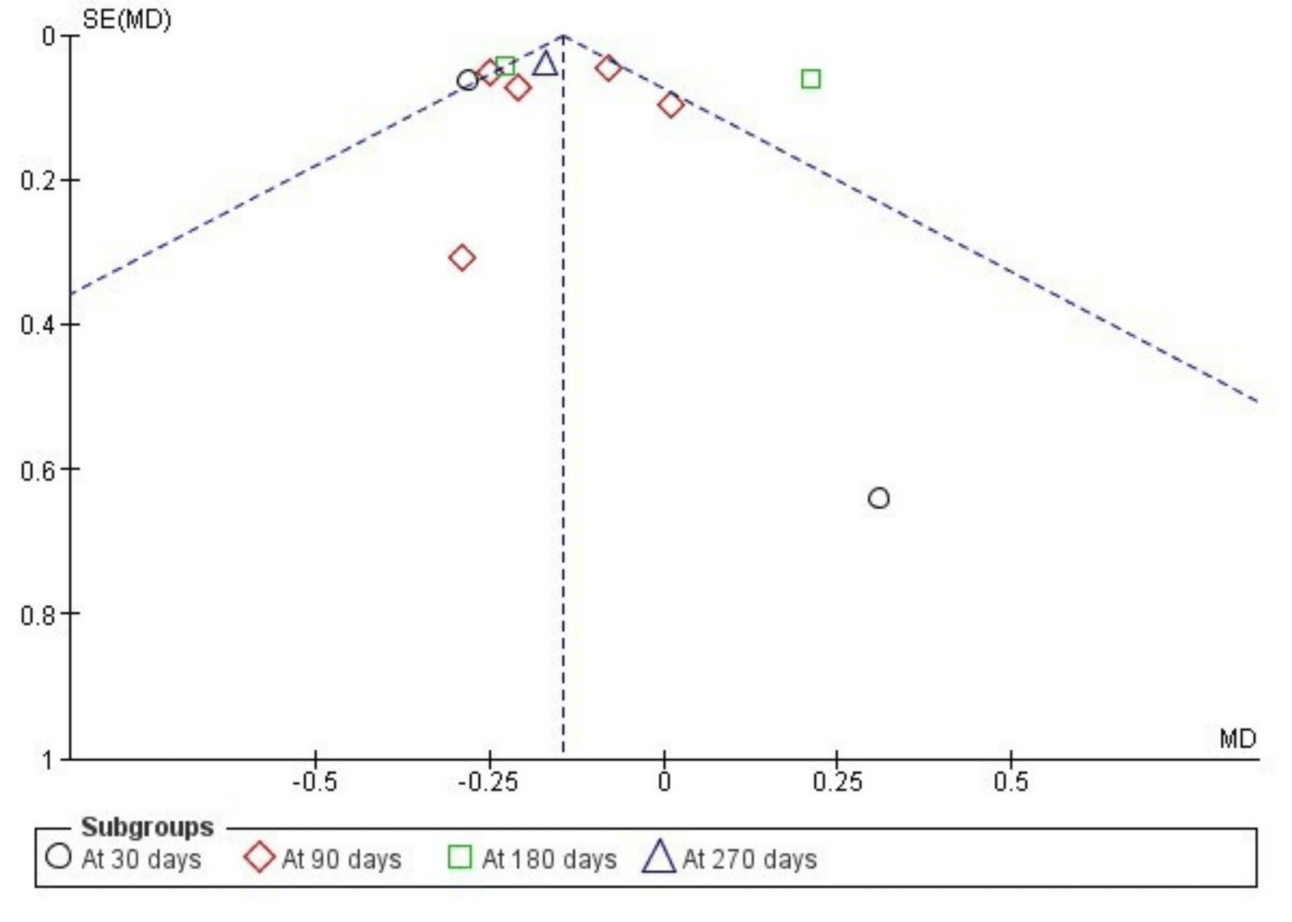
Funnel plot showing publication bias in the assessment of gingival index (indicating studies with high standard error and wide confidence interval)

The funnel plot for comparison of ATP and other methods revealed a moderately suspected publication bias in all PI and GI assessments with high standard error between the samples and the actual population (Figure 7 and Figure 8).

Certainty of Evidence

Certainty of evidence assessed using GRADEpro reported moderate to high levels of certainty for plaque and GI at different follow-up periods (Table 5 and Table 6).

**Table 5:** GRADEpro assessment of the certainty of the evidence of plaque scores as continuous data ATP: audio-tactile performance; MD: mean difference

No. of studies	Study design	Risk of bias	Inconsistency	Indirectness	Imprecision	Other considerations	No. of patients - ATP	No. of patients - Braille/others	Relative effect (95% CI)	Absolute effect (95% CI)	Certainty	Importance
Plaque score (At 30 days)												
1	Non-randomized trial	Not serious	Not serious	Not serious	Not serious	None	30	30	-	MD -0.27 higher (-0.39 higher to -0.15 higher)	⨁⨁⨁⨁ High	Important
Plaque score (at 90 days)												
5	Randomized trial	Serious	Not serious	Not serious	Not serious	Publication bias	193	193	-	MD -0.03 higher (-0.08 higher to 0.03 higher)	⨁⨁⨁ Moderate	Important
Plaque score (at 180 days)												
2	Randomized trial	Serious	Not serious	Not serious	Not serious	None	60	60	-	MD -0.10 higher (-0.17 higher to 0.02 higher)	⨁⨁⨁ Moderate	Important
Plaque score (at 270 days)												
1	Non-randomized trial	Serious	Not serious	Not serious	Not serious	None	30	30	-	MD -0.17 higher (-0.25 higher to -0.09 higher)	⨁⨁⨁ Moderate	Important
Plaque score (total)												
5	Randomized trial	Serious	Not serious	Not serious	Not serious	None	343	343	-	MD -0.10 higher (-0.13 higher to 0.06 higher)	⨁⨁⨁ Moderate	Important

**Table 6:** GRADEpro assessment of the certainty of the evidence of gingival scores as continuous data ATP: audio-tactile performance; MD: mean difference

No. of studies	Study design	Risk of bias	Inconsistency	Indirectness	Imprecision	Other considerations	No. of patients - ATP	No. of patients - Braille/others	Relative effect (95% CI)	Absolute effect (95% CI)	Certainty	Importance
Gingival score (at 30 days)												
2	Randomized trial	Not serious	Not serious	Not serious	Not serious	Publication bias	60	60	-	MD -0.27 higher (-0.41 higher to -0.15 higher)	⨁⨁⨁⨁ High	Important
Gingival score (at 90 days)												
5	Randomized trial	Serious	Not serious	Not serious	Not serious	Publication bias	195	195	-	MD -0.15 higher (-0.21 higher to 0.09 higher)	⨁⨁⨁ Moderate	Important
Gingival score (at 180 days)												
2	Randomized trial	Serious	Not serious	Not serious	Not serious	None	62	62	-	MD -0.09 higher (-0.15 higher to -0.02 higher)	⨁⨁⨁ Moderate	Important
Gingival score (at 270 days)												
1	Non-randomized trial	Serious	Not serious	Not serious	Not serious	None	30	30	-	MD -0.17 higher (-0.25 higher to -0.09 higher)	⨁⨁⨁ Moderate	Important
Gingival score (total)												
6	Randomized trial	Serious	Not serious	Not serious	Not serious	None	343	343	-	MD -0.15 higher (-0.18 higher to 0.11 higher)	⨁⨁⨁ Moderate	Important

Discussion

This systematic review has yielded nine studies [13-21] that compared the ATP method with other health education methods for educating visually impaired children on oral health. The studies that have been included in this review, although few in number, had varied results. However, this review has summarized the contemporary evidence available and will lay the foundation for future research.

Transfer of knowledge and skills as a preventive approach to oral disease, thus improving the quality of life of an individual, group, or community, is referred to as OHE. These educational programs focus on teaching and encouraging the beneficiary to maintain optimal oral health followed by regular reinforcement to ultimately result in a holistic and comprehensive method of maintaining a healthy body and mind [17].

As children learn and model habits very early in their lives, repeated reinforcement and practice at an early age will help the child inculcate the habit with greater efficiency and also help them retain it throughout their adult life [22].

Visual perception is the major element contributing to learning in traditional health education methods, such as the use of disclosing solutions to reveal plaque. However, these techniques are not suitable for visually impaired children as they rely on senses other than vision such as feeling and hearing [23].

Individuals with visual impairment have unacceptable oral health attributable to their inability to visualize plaque formation and detect early signs of caries formation such as discoloration [24].

Due to their inability to visualize their surroundings, blind individuals orient themselves to the world using their other senses such as touch, smell, and hearing. The process of imparting oral hygiene measures and techniques to blind children mandates the need for a different and innovative approach encompassing patience and sufficient time with tactile sensation as the foundation [9].

A combination method called ATP first surfaced in the literature in 2012, and there are increasing numbers of studies investigating the effectiveness of this technique [25,26]. This technique has three parts: (1) Audio: Interaction with the children to ensure the establishment of sound rapport with the dentist while maintaining a friendly environment conducive to learning. (2) Tactile: Children are made to feel the various parts of the oral cavity on a tooth model that is large in size. The children are also instructed to use their tongues and look for deposits, which would be indicated by a feeling of roughness. It is followed by making them feel their teeth on a large-sized model (tactile). (3) Performance: The children are first instructed to implement the brushing technique on the tooth model, after which they are instructed to brush their own teeth with the necessary assistance [27].

In research done by Diptajit Das et al. [13], OHE was imparted using a combination of Braille and audio aids (control) and was compared with the ATP technique (test). Post-intervention, children in the test arm (13.73 ± 2.71) had better knowledge than those in the control (9.56 ± 2.19) arm, which was statistically significant (p < 0.05). For assessing the reduction in plaque levels between the groups, the plaque scores after brushing were subtracted from the plaque scores before brushing. A greater lowering of plaque levels was seen in the test arm (2.75 ± 1.76) when compared with the control arm (2.63 ± 2.02) at baseline, though this did not have a statistically significant difference. Similarly, a statistically insignificant difference was observed between the test and control groups at 90 days; the plaque reduction was higher in the test group (3.5 ± 1.18) than in the control group (3.14 ± 0.88). Though the test group had better outcomes with respect to knowledge score, PI, and GI, the difference was not significant. Hence, ATP was concluded to be equally effective as the other methods. The authors attributed this to the concept of Braille and audio methods being more commonly followed in the children’s daily lives, ultimately leading to familiarity and, hence, improved recollection.

However, in contrast, in a study carried out by Deolia et al., there was a statistically significant decrease in the post-interventional (Fones brushing technique taught using ATP) plaque scores from 2.78 to 1.63 (p < 0.05) and an increase in the post-health education test scores from 1.98 to 8.21 (p < 0.05) [28].

Similar observations were made in research by Ganapathi et al. [29]. This increase in plaque reduction could be due to the disciplined and organized lifestyle followed by the children in the residential school, which enabled them to be more receptive to understanding new concepts. As the ATP method enhances the working of the cognitive centers, OHE concepts would have been better recalled by the children after the intervention.

In the study conducted by Rupali Shrivastava et al. [20], the verbal method (group 1), Braille method (group 2), and ATP method (group 3) were compared. The scores for debris index (DI), calculus index (CI), and GI at six months were lowest in group 2, and the difference when compared to other groups was statistically significant. Hence, it was concluded that the Braille method was superior to the other methods in its effectiveness in improving oral hygiene. This was in accordance with studies conducted by Deshpande et al. [13] and Ganapathi et al. [29], who found that many students reached the good score category after being initially classified as fair following the use of the Braille technique. This could be attributed to Braille usually being applied in their daily routine and being perceived in a better way as compared to the novel technique given in the ATP group.

In the study conducted by Kompal Gautham et al. [17], Braille (group 1), ATP (group 2), and a combination of ATP and Braille (group 3) were compared. There was a highly significant difference seen for the intergroup comparison of post-PI (p < 0.01) and post-GI (p < 0.01) with the least mean in group 3. There was a statistically highly significant difference seen for the intra-group comparison of pre- and post-PI and GI (p < 0.01) with lesser means in post-PI as compared to pre-PI in all three groups. They concluded that a combination of Braille and ATP was required to improve the oral health of the children. This is in accordance with a study conducted by Nair et al. [19], where it was observed that the ATP + Braille technique resulted in a greater reduction in plaque scores during both the reinforcement and non-reinforcement periods than the ATP technique alone. The authors supported this finding by adding that the inclusion of Braille may have helped to convert the tactile sensations into learned information. Moreover, as the Braille pamphlet included a story, it may have been more appealing to the children and, therefore, better remembered.

In the study conducted by Nasrin Sharififard et al., the art group (ATP, game-based, and music-based education), the mothers group (children received ATP and their mothers received education by telephone), and the control group (children received ATP) were compared. The art and mothers groups had no statistically significant difference compared with the control group in terms of OHI-S. Hence, the authors concluded that ATP alone was sufficient to improve the oral health of the children and suggested that hand-over-hand guidance for teaching brushing provided a proper tactile sense and positive emotional feeling. Their perfect consequent performance of tooth brushing made them confident to do it at home. This is in line with Hebbal’s study [26] that showed pre- and post-education through ATP decreased mean plaque scores significantly (p < 0.001).In a similar study conducted by Shetty et al. [30], OHE was imparted with the help of specially designed models and music-aided instructions in a song format, resulting in a significant improvement in the oral health status of blind children. This is also in line with a study conducted by Gautam et al. [17]; audio + Braille, audio + tactile, and audio + Braille + tactile groups were compared for their patient hygiene performance index score, and the difference was not statistically significant.

In the most recent study conducted in 2024, Santhoshi et al. compared Braille with NonVisual Desktop Access (NVDA) for OHE. They observed that the mean PI scores and mean OHI-S scores were higher in the Braille group compared to the NVDA group [10].

Future studies should consider blinding the educator and examiner or using methods that minimize subjectivity (such as plaque indicating dye, assessment using clinical photographs, or triangulation with multiple examiners). Other investigations that could be of interest include the effect of recall intervals, age, literary ability, and degree of visual impairment on the acceptance magnitude of change. Moreover, data on the retention of the health education received even after discontinuation of intervention is a knowledge gap that must be bridged. Studies need to be conducted to discuss how the methods used in OHE for visually impaired children can describe the motivation of the professional, his ability to establish a good communication channel with the child and their parents, demonstrate empathy, and factors related to the cultural context.

Limitations

A high risk of bias was seen in the included studies. There was also limited reporting on the methods, such as how the randomization and blinding were performed and how the interventions were delivered.

## Conclusions

Though ATP exhibited better improvement in the oral health of visually impaired children when compared to other conventional methods, the studies included high levels of heterogeneity. Future research should work on controlling the factors that have been identified as causing a high risk of bias in this study. Areas that could be improved include pre-trial registration with a well-designed and transparent plan and reporting according to the Consolidated Standards of Reporting Trials (CONSORT) guidelines.
